# Plasma concentration of *cis*9*trans*11 CLA in males and females is influenced by *SCD1* genetic variations and hormonal contraceptives: a cross-sectional study

**DOI:** 10.1186/1743-7075-10-50

**Published:** 2013-07-18

**Authors:** Salma A Abdelmagid, Shannon E Clarke, Jeremy Wong, Kaitlin Roke, Daiva Nielsen, Alaa Badawi, Ahmed El-Sohemy, David M Mutch, David WL Ma

**Affiliations:** 1 Department of Human Health and Nutritional Sciences, Animal Science/Nutrition Building, Rm 342, College of Biological Science, University of Guelph, 491 Gordon Street, Guelph, Ontario, Canada; 2 Department of Nutritional Sciences, University of Toronto, Toronto, Ontario, Canada; 3 Office for Biotechnology, Genomics and Population Health, Public Health Agency of Canada, Toronto, Ontario, Canada

**Keywords:** c9t11 CLA, SCD1, Hormonal contraceptives, Single nucleotide polymorphisms

## Abstract

**Background:**

The conjugated linoleic acid isomer cis9trans11 CLA can be endogenously synthesized from trans vaccenic acid (C18:1 t11) via desaturation at the delta 9 position catalyzed by the stearoyl-CoA desaturase 1 (SCD1), also known as delta-9 desaturase (D9D). Diet, hormonal regulation of gene expression and single nucleotide polymorphisms (SNPs) have been implicated in altering circulating levels of fatty acids. Hormonal contraceptives (HC) have also been shown to influence levels of some fatty acids. SNPs in *SCD1* have been associated with altered levels of palmitoleic and oleic acids; however, associations between *SCD1* SNPs and D9D desaturation index have not been previously examined in relation to CLA. Herein, we investigated the effects of sex and HC use on circulating concentrations of c9t11 CLA and D9D desaturation index. Furthermore, we determined the effects of ten *SCD1* SNPs on D9D desaturation indices estimated by product to precursor ratio of c9t11 CLA to C18:1 t11.

**Methods:**

Plasma samples were collected from subjects (Caucasian males: n = 113; Caucasian females: n = 298; Asian males: n = 98; Asian females: n = 277) from the Toronto Nutrigenomics and Health Study. Circulating fatty acids levels were measured by gas chromatography.

**Results:**

Results show that circulating c9t11 CLA concentrations are significantly higher in females than males and they are further elevated in females using HC. In addition, a significant sex- and ethnic-specific association was found between *SCD1* SNP rs10883463 (p = 0.0014) and altered D9D activity in Caucasian males.

**Conclusion:**

Findings from the present study identify *SCD1* SNPs and hormonal contraceptives as factors altering endogenous c9t11 CLA levels in a sex- and ethnic-specific manner.

## Background

Conjugated linoleic acids (CLA) are 18 carbon polyunsaturated fatty acids that are naturally found in ruminant meat and milk products. CLA isomers contain conjugated double bonds in different cis and trans configurations. CLA isomers include trans10cis12 CLA, cis11trans13 CLA and the most common isomer cis9trans11 CLA (c9t11 CLA), also known as rumenic acid (RA) [1]. RA is the primary CLA isomer in ruminant and dairy products where it constitutes more than 80% of total CLA [2-4]. Considerable attention has been given to CLA as it has been shown to possess anti-carcinogenic, anti-atherosclerotic and anti-inflammatory properties [5-13].

CLA is produced through biohydrogenation of polyunsaturated fatty acids by ruminant bacteria [14]. In addition, the CLA isomer c9t11 can also be derived through the delta-9-destaturation of the ruminant trans vaccenic acid (C18:1 t11). Trans vaccenic acid is the most abundant trans fatty acid (TFA) in ruminant meat and dairy products, although lesser amounts are also produced industrially [3,15]. Ruminant TFA are suggested to confer health benefits; however, more research is needed to substantiate their beneficial effects [16]. In a study conducted in male Hartley guinea pigs, animals fed ruminant TFA compared to animals fed industrial TFA had a smaller HDL particle profile; which has been associated with a lower risk of cardiovascular and heart disease [17]. Using a rodent model of metabolic syndrome, Wang et al. have shown trans vaccenic acid to have beneficial effects on lipids and lipoproteins [18]. Furthermore, trans vaccenic acid was shown to decrease the proinflammatory markers IL-2 and TNF-α [19,20].

In humans, trans vaccenic acid is converted to c9t11 CLA through a desaturation reaction catalyzed by stearoyl-CoA desaturase 1 (SCD1), also known as delta-9 desaturase (D9D) [21-25]. SCD1 is a membrane-bound 40 kDa protein localized to the endoplasmic reticulum [23]. It introduces a cis double bond in the delta 9 position of fatty acyl-CoA substrates, preferably palmitoyl-CoA (C16:0) and stearoyl-CoA (C18:0) to produce palmitoleoyl-CoA (C16:1) and oleoyl-CoA (C18:1), respectively. Changes in *SCD1* expression have been implicated in several diseases such as skin inflammation, pancreatic β-cell dysfunction, liver dysfunction and atherosclerosis [26]. Factors contributing to changes in *SCD1* expression and SCD1 activity include hormonal regulation and single nucleotide polymorphisms (SNPs) [27,28]. Sex differences in *SCD1* expression were demonstrated in mice where females had higher levels of expression accompanied by higher levels of palmitoleic and oleic acids in the liver [29]. However, the underlying mechanistic basis for those differences is unknown. In humans, sex differences in *SCD1* expression or levels of delta-9 desaturation product c9t11 CLA are not yet determined. Hormonal contraceptives (HC) have been shown to influence levels of the fatty acid docosahexaenoic acid which have also been shown to be present in significantly higher levels in females than in males [30]. Effect of HC use on the delta-9 desaturation index or c9t11 CLA levels is unknown.

SNPs in *SCD1* have been associated with decreased body mass index (BMI), waist circumference and improved insulin sensitivity in elderly Swedish men [31]. Recently, SNPs in *SCD1* have been shown to influence plasma levels of 16:0, 16:1, and 18:0, as well as C-reactive protein, in individuals of European descent [28]; however, the effect of *SCD1* genetic variations on c9t11 CLA concentrations has not been previously examined. Consequently, it is unknown whether *SCD1* genetic variants result in sex differences in circulating concentrations of c9t11 CLA. Therefore, the present study examined the influence of *SCD1* genetic variation and HC on circulating concentrations of c9t11 CLA in men and women in Canadian population of Caucasians and Asians.

## Methods

### Study population

Participants (Caucasians: males = 113, females = 298; Asians: males = 98, females = 277) were recruited as part of the Toronto Nutrigenomics and Health (TNH) Study [32] between September 2004 and July 2009. Ages of participants ranged between 20 and 29 years old and written informed consent was obtained from all of those who participated. Anthropometric measurements were recorded for all participants and health, life style, and food frequency questionnaires were completed by subjects. Total energy intake from fat and physical activity scores were calculated from completed questionnaires as previously described [32,33]. Following a 12 h fast, plasma samples were collected for measurement of biomarkers of glucose and lipid metabolism. HOMA-IR was calculated using the homeostasis model assessment method [34]. Women who were pregnant or breastfeeding were not included in the study. No other exclusion criteria were included in these analyses. The study protocol was approved by the Research Ethics Boards at the University of Toronto and University of Guelph.

### Gas chromatography analysis

Subjects were required to fast overnight for a minimum of 12 h prior to blood collection and separation of plasma. Samples were frozen and stored at - 80°C. Frozen plasma samples were thawed on ice for 30 min and a mixture of chloroform: methanol (2:1 v/v) was added to a 50 μl aliquot as described previously [35]. Free fatty acid C17:0 was used as an internal standard (5 μg of 1 mg/ml stock). Samples were flushed with nitrogen gas prior to storage over night at 4°C. The next day, samples were subjected to a double extraction, saponification, methylation and quantification of fatty acid methyl esters, as previously described [36]. Fatty acid methyl esters were separated by gas chromatography using an Agilent7890A gas chromatograph (Agilent Technologies, Palto, Alto, Ca) with a Supelco SP 2560 fused-silics capillary column (100 m × 0.25 mm i.d., 0.2 μm film thickness; Sigma-Aldrich, St-Louis, MO). The carrier gas, Hydrogen, was set at a constant flow rate of 30 mL/min. Samples were injected in splitless mode. Both injector and detector ports were set at 250°C. Fatty acid methyl esters were eluted through the column using temperature program of: 0.2 min at 60°C, then increasing 13°C/min until a temperature of 170°C was reached. After 4 min at 170°C, the temperature: increased 6.5°C/min to 175°C, increased 2.6°C/min to 185°C, increased 1.3°C/min to 190°C, and finally increased 13°C/min to 240°C and held at 240°C for 13 min. The processing time per sample was 37.77 min. Peak retention times of a known internal standard (Nu-Chek-Prep, Elysian, MN) were used to identify fatty acids. Fatty acid peak areas were determined using EZChrom Elite software (Version 3.3.2) [37]. The internal standard was used to calculate fatty acid concentrations (μg/ml). An estimate of delta-9-desaturase (D9D) desaturation index was calculated by dividing concentrations of product (18:2c9t11 CLA) by precursor (18:1 t11) as previously described [36].

### Genotyping

Identification of *SCD1* SNPs, selection of SNPs, and genotyping was performed as previously described by Stryjecki et al. [28].

### Statistical analysis of data

Results are expressed as mean ± standard error mean. All data was analyzed using JMP genomics software V5 (SAS Institute, Cary, NC), which was also used to test Hardy-Weinberg equilibrium for each genotype. A student’s t-test was used to determine differences in fatty acid concentrations and ratios between males and females. A Tukey’s HSD post-hoc test was used to determine differences in desaturase indices for each genotype. P-values of analyses of fatty acid levels or desaturase indices were determined using linear regression models which were adjusted for BMI, age, total energy intake from dietary fat and physical activity. Linear regression models were used to identify associations between individual SNPs, desaturase indices and use of hormonal contraceptives and were adjusted for BMI, age, % of Total Energy from Dietary Fat and physical activity. A p-value of < 0.05 was considered statistically significant.

## Results

### Study population

General characteristics of subjects used in the study are presented in Table 1.

**Table 1:** General characteristics of study population at fasting state compared by sex and separated by ethnicity

	**Caucasians**			**Asians**		
	**Males**	**Females**	**P-value**	**Males**	**Females**	**P-value**
Population (#)	113	298		98	277	
Hormonal contraceptive users (%)	N/A	46.0		N/A	15.5	
Age (yrs)	23.1 ± 0.2	23.1 ± 0.1	0.87	22.4 ± 0.2	22.1 ± 0.1	0.26
BMI (kg/m^2^)	23.3 ± 0.3	23.1 ± 0.2	0.54	23.2 ± 0.3	21.2 ± 0.1	<0.01*
HOMA-IR	1.16 ± 0.08	1.39 ± 0.05	0.02*	1.42 ± 0.08	1.45 ± 0.10	0.84
Glucose (mmol/L)	4.88 ± 0.07	4.70 ± 0.02	<0.01*	4.95 ± 0.04	4.73 ± 0.02	<0.01*
Insulin (pmol/L)	37.1 ± 2.1	47.4 ± 1.7	<0.01*	45.7 ± 2.3	48.1 ± 2.9	0.62
Total cholesterol (mmol/L)	3.97 ± 0.07	4.35 ± 0.05	<0.01*	4.13 ± 0.08	4.29 ± 0.04	<0.05*
HDL-cholesterol (mmol/L)	1.38 ± 0.03	1.69 ± 0.02	<0.01*	1.39 ± 0.03	1.68 ± 0.02	<0.01*
LDL-cholesterol (mmol/L)	2.16 ± 0.06	2.23 ± 0.04	0.32	2.29 ± 0.07	2.21 ± 0.04	0.24
Triglycerides (mmol/L)	0.96 ± 0.05	0.97 ± 0.02	0.86	0.98 ± 0.05	0.91 ± 0.02	0.19
Free fatty acids (mmol/L)	454 ± 24.6	489 ± 14.2	0.21	453 ± 21.4	521 ± 15.4	0.02*
% of Total Energy from Dietary Fat	27.4 ± 0.6	27.9 ± 0.4	0.56	26.3 ± 0.5	26.2 ± 0.3	0.98

Statistical differences were determined using a Student’s T-test. The * denotes p-values which are significant (< 0.05).

### Gender influences plasma concentrations of 18:2c9t11 and D9D index

Plasma concentrations of fatty acids are presented in Table 2. There was no significant difference in 18:1 t11 concentrations between Caucasian males and females; however, Asian females had significantly higher concentrations of 18:1 t11 compared to Asian males (p = 0.04). Both Caucasian and Asian females had significantly higher concentrations of 18:2c9t11 and D9D indices compared to males within the same ethnicity (p < 0.01).

**Table 2:** Estimates of fatty acid concentrations (μg/ml) in Caucasian and Asian males and females

**Fatty acids**	**Caucasians**			**Asians**		
	**Males**	**Females**	**P-value**	**Males**	**Females**	**P-value**
18:0	131.9 ± 3.3	137.7 ± 1.8	0.08	133.1 ± 3.0	140.1 ± 1.8	0.03*
18:1 t9	4.1 ± 0.2	4.7 ± 0.2	0.04*	3.7 ± 0.2	4.4 ± 0.2	<0.01*
18:1 t11	4.6 ± 0.2	4.8 ± 0.2	0.50	4.0 ± 0.2	4.3 ± 0.2	0.04*
18:2c9t11	4.5 ± 0.2	5.3 ± 0.1	<0.01*	3.7 ± 0.1	4.1 ± 0.1	<0.01*
18:2c11t13	0.5 ± 0.0	0.6 ± 0.0	0.15	0.5 ± 0.1	0.5 ± 0.0	<0.01*
18:2t10c12	1.1 ± 0.1	1.2 ± 0.0	0.16	1.2 ± 0.1	1.2 ± 0.0	0.73
18:2c9t11/18:1 t11	1.0 ± 0.0	1.2 ± 0.0	<0.01*	1.1 ± 0.0	1.1 ± 0.0	0.01*

Select fatty acids used as substrate and product of D9D, desaturase index and major CLA isomers are reported. The * denotes p-values which are significant (< 0.05). P-values were determined using linear regression models which were adjusted for BMI, age, % of Total Energy from Dietary Fat and physical activity. Caucasian Males: n = 113; Caucasian females: n = 298; Asian Males: n = 98; Asian Females: n = 277.

### Association of hormonal contraceptive (HC) use and altered fatty acid concentrations

Plasma concentrations of fatty acids in females using hormonal contraceptives (HC) compared to females not using contraceptives are presented in Table 3. In Caucasians, females using HC had significantly higher concentrations of 18:1 t11 and 18:2c9t11 than Caucasian females who were not users (p = 0.0037, p < 0.0001, respectively). In Asians, there was no significant difference in 18:1 t11 concentrations between users and non-users; however, females using HC had significantly higher concentrations of 18:2c9t11 (p < 0.0001). In both Caucasians and Asians, [18:2c9t11/18:1 t11] desaturase indices tended to be higher in females using HC (p = 0.0581 and p = 0.0503, respectively) than in females not using contraceptives (Table 3).

**Table 3:** Estimates of fatty acid concentrations (μg/ml) in Caucasian and Asian females using hormonal contraceptives

**Fatty acids**	**Caucasians**			**Asians**		
	**non (n = 161)**	**HC (n = 137)**	**P-value**	**non (n = 234)**	**HC (n = 43)**	**P-value**
18:0	134.7 ± 2.5	141.3 ± 2.5	0.03	139.4 ± 2.0	143.6 ± 4.2	0.20
18:1 t9	4.2 ± 0.2	5.2 ± 0.3	<0.01*	4.2 ± 0.2	5.5 ± 0.5	0.02*
18:1 t11	4.4 ± 0.2	5.3 ± 0.3	<0.01*	4.2 ± 0.2	5.0 ± 0.4	0.13
18:2c9t11	4.5 ± 0.1	6.1 ± 0.2	<0.01*	3.9 ± 0.1	5.2 ± 0.3	<0.01*
18:2c11t13	0.6 ± 0.0	0.6 ± 0.0	0.07	0.5 ± 0.0	0.7 ± 0.1	<0.01*
18:2t10c12	1.1 ± 0.1	1.2 ± 0.0	0.12	1.2 ± 0.0	1.3 ± 0.1	0.26
18:2c9t11/18:1 t11	1.2 ± 0.1	1.3 ± 0.0	0.06	1.1 ± 0.0	1.2 ± 0.1	0.05

Select fatty acids used as substrate and product of D9D, desaturase index and major CLA isomers are reported. *denotes p-values which are significant (< 0.05). P-values were determined using linear regression models which were adjusted for BMI, age, % of Total Energy from Dietary Fat and physical activity. Abbreviations: *HC*, females using hormonal contraceptives; non, females that are not using hormonal contraceptives.

### SNP selection

The 10 selected SNPs from the *SCD1* gene were in HWE in Caucasian males and females as well as in the Asian females (Table 4). *SCD1* SNP: rs575338 was not polymorphic (MAF = 0) in Asian males and, therefore, was excluded from further analyses in this subgroup of subjects (Table 4).

**Table 4:** **HWE values of *SCD1 *and their genotype frequencies**

**SNP**	**Caucasians**						**Asians**					
	**Males**			**Females**			**Males**			**Females**		
	**HWE**	**Major allele**	**MAF**	**HWE**	**Major allele**	**MAF**	**HWE**	**Major allele**	**MAF**	**HWE**	**Major allele**	**MAF**
rs10883463	0.79	T	0.09	0.99	T	0.10	0.99	T	0.01	0.98	T	1.9•10^-3^
rs417669878	0.79	G	0.42	0.99	G	0.42	0.98	G	0.26	0.98	G	0.28
rs2060792	0.83	A	0.26	0.99	A	0.29	0.99	A	0.39	0.98	A	0.31
rs3071	0.79	T	0.29	0.99	T	0.31	0.98	T	0.27	0.98	T	0.28
rs3793766	0.79	C	0.12	0.99	C	0.08	0.99	C	0.17	0.98	C	0.18
rs3793767	0.82	T	0.40	0.99	T	0.37	0.98	T	0.45	0.98	T	0.35
rs490726	0.67	T	0.22	0.99	T	0.19	0.99	T	0.23	0.98	T	0.28
rs522951	0.83	G	0.48	0.99	G	0.49	0.99	G	0.42	0.98	G	0.32
rs569184	0.79	C	0.12	0.99	C	0.08	0.99	C	0.20	0.98	C	0.28
rs575338	0.79	G	0.15	0.99	G	0.13	NP	G		0.98	G	5.6•10^-3^

SNPs with HWE p-values less than 0.05 are not in Hardy-Weinberg Equilibrium. Caucasian Males: n = 113; Caucasian females: n = 298; Asian Males: n = 98; Asian Females: n = 277. Abbreviations: *HWE*, Hardy-Weinberg Equilibrium p-values; *MAF*, minor allele frequency; *NP*, not polymorphic (MAF = 0).

### Association of *SCD1* SNPs and [18:2c9t11/18:1 t11] desaturase indices

Regression analysis of ten *SCD1* SNPS and D9D indices [18:2c9t11/18:1 t11] revealed no significant associations in Caucasian females, Asian males, or Asian females (Tables 5 and 6). However, in Caucasian males rs10883463 was significantly associated with altered D9D desaturation index (p < 0.01). In this population, carriers of the minor C allele (19 out of 113 Caucasian males) had significantly lower D9D desaturase indices compared to homozygote carriers of the common T allele (Table 5). Further analysis to determine associations between rs10883463 and other D9D desaturation indices ([18:1/18] and [16:1/16]) revealed no significant associations (data not shown). Thus, the association identified between rs10883463 and [18:2c9t11/18:1 t11] desaturase index potentially reveals a substrate specific association between this SNP and D9D enzymatic activity; however, this association was lost when we adjusted for multiple testing.

**Table 5:** **Estimates of D9D activity according to genotype for each *SCD1 *SNP in Caucasians**

	**[18:2c9t11/18:1 t11] in males**				**[18:2c9t11/18:1 t11] in females**			
	**MM**	**Mm**	**mm**	**P-value**	**MM**	**Mm**	**mm**	**P-value**
rs10883463	1.077 ± 0.036^a^	0.769 ± 0.048^b^	-	<0.01*	1.206 ± 0.036	1.244 ± 0.061	1.505 ± 0.170	0.75
rs17669878	1.079 ± 0.070	0.977 ± 0.040	1.064 ± 0.076	0.23	1.248 ± 0.041	1.195 ± 0.050	1.207 ± 0.074	0.72
rs2060792	1.015 ± 0.037	0.998 ± 0.061	1.215 ± 0.179	0.62	1.251 ± 0.053	1.175 ± 0.036	1.229 ± 0.088	0.58
rs3071	1.037 ± 0.052	1.024 ± 0.046	0.913 ± 0.051	0.78	1.202 ± 0.031	1.224 ± 0.059	1.238 ± 0.105	0.87
rs3793766	1.003 ± 0.036	1.083 ± 0.078	1.200	0.59	1.217 ± 0.035	1.209 ± 0.057	1.084 ± 0.391	0.94
rs3793767	0.939 ± 0.042	1.043 ± 0.051	1.111 ± 0.097	0.25	1.178 ± 0.043	1.252 ± 0.052	1.200 ± 0.054	0.81
rs490726	1.073 ± 0.042	0.960 ± 0.053	0.856 ± 0.344	0.23	1.215 ± 0.041	1.217 ± 0.046	1.229 ± 0.107	0.99
rs522951	1.044 ± 0.046	1.011 ± 0.050	1.019 ± 0.081	0.78	1.125 ± 0.055	1.252 ± 0.049	1.237 ± 0.048	0.42
rs569184	1.010 ± 0.036	1.060 ± 0.079	1.200	0.72	1.223 ± 0.035	1.198 ± 0.054	0.637 ± 0.055	0.38
rs575338	1.024 ± 0.042	1.024 ± 0.045	1.003 ± 0.155	0.99	1.193 ± 0.029	1.291 ± 0.098	1.188 ± 0.084	0.44

Results of analysis of 10 SNPs from *SCD1* for associations with D9D activity index [182c9t11:181 t11]. Different letters (a or b) denote values that are significantly different between groups. P-values were determined using linear regression models which were adjusted for BMI, age, % of Total Energy from Dietary Fat and physical activity. Caucasian Males: n = 113; Caucasian females: n = 298; Asian Males: n = 98; Asian Females: n = 277. Abbreviations: *D9D*, delat-9-desaturase; *M*, major; m, minor; ‘-’ indicates that no subjects with this genotype were present in this population.

**Table 6:** **Estimates of D9D activity according to genotype for each *SCD1 *SNP in Asians**

	**[18:2c9t11/18:1 t11] in males**				**[18:2c9t11/18:1 t11] in females**			
	**MM**	**Mm**	**mm**	**P-value**	**MM**	**Mm**	**mm**	**P-value**
rs10883463	1.039 ± 0.041	0.813 ± 0.064	-	0.46	1.085 ± 0.027	0.783	-	0.23
rs17669878	1.094 ± 0.059	0.966 ± 0.052	0.910 ± 0.118	0.18	1.035 ± 0.035	1.139 ± 0.043	1.061 ± 0.096	0.28
rs2060792	0.949 ± 0.049	1.052 ± 0.064	1.185 ± 0.107	0.27	1.090 ± 0.040	1.058 ± 0.038	1.185 ± 0.084	0.37
rs3071	1.069 ± 0.061	0.959 ± 0.048	1.103 ± 0.121	0.34	1.052 ± 0.038	1.143 ± 0.041	0.939 ± 0.063	0.11
rs3793766	1.035 ± 0.047	1.053 ± 0.081	0.752 ± 0.036	0.88	1.072 ± 0.030	1.117 ± 0.056	0.906 ± 0.081	0.59
rs3793767	0.993 ± 0.057	1.028 ± 0.058	1.120 ± 0.104	0.66	1.109 ± 0.044	1.051 ± 0.037	1.120 ± 0.067	0.54
rs490726	1.035 ± 0.048	1.070 ± 0.077	0.795 ± 0.051	0.68	1.056 ± 0.034	1.128 ± 0.044	1.038 ± 0.101	0.19
rs522951	0.975 ± 0.053	1.030 ± 0.062	1.164 ± 0.102	0.40	1.095 ± 0.041	1.058 ± 0.038	1.156 ± 0.080	0.42
rs569184	1.041 ± 0.049	1.052 ± 0.076	0.807 ± 0.064	0.86	1.045 ± 0.034	1.129 ± 0.046	1.070 ± 0.093	0.25
rs575338	N/A				1.082 ± 0.027	0.815 ± 0.143	-	0.47

Results of analysis of 10 SNPs from *SCD1* for associations with D9D activity index [182c9t11:181 t11]. P-values were determined using linear regression models which were adjusted for BMI, age, % of Total Energy from Dietary Fat and physical activity. Caucasian Males: n = 113; Caucasian females: n = 298; Asian Males: n = 98; Asian Females: n = 277. Abbreviations: *D9D*, delat-9-desaturase; *M*, major; *m*, minor; *na*, not applicable; ‘-’ indicates that no subjects with this genotype were present in this population.

## Discussion

Circulating levels of c9t11 CLA may be potentially influenced by a number of factors including differences in dietary intake [38,39], differential expression of *SCD1*, or genetic variations in *SCD1* leading to differential enzymatic activity. The latter two factors have not been previously examined and are the focus of the present study where we show sex-specific modulation of circulating CLA.

Firstly, plasma concentrations of c9t11 CLA in two major ethnicities and both sexes were quantified on an absolute basis. These results revealed significant differences in circulating plasma concentrations of c9t11 CLA between males and females in both ethnicities. Females had significantly higher circulating c9t11 concentrations than their male counterparts (p < 0.01). Previously, Zlatanos et al. reported plasma CLA levels in humans but as percent of total fatty acids and although both males and females were included in the study, comparison of CLA levels between sexes was not performed [39]. In the present study plasma concentrations of fatty acids were determined because they provide a quantitative perspective on the pool size as compared to relative percent composition, which is influenced by the abundance of other fatty acids. Others have determined plasma concentrations of CLA in dietary intervention studies [38,40,41] which fall within the same range found in this study (0.5 - 40 μmol/L). Herbel et al. included both males and females in their study of the effects of sunflower consumption on circulating CLA concentrations [40]; however, comparison of circulating c9t11 CLA concentrations between males and females or different ethnicities was not performed. We recognize that differences in CLA concentrations, determined in this study, between males and females may be viewed as small. Whether these apparent small changes are relevant to health will require further human studies. Nonetheless, these data contribute to our fundamental knowledge of baseline levels of CLA, and factors influencing these levels, which will be necessary for such studies. Our study participants were not consuming a standardized diet; however, values for energy intake from fat (i.e. % of Total Energy from Dietary Fat) were calculated with food frequency questionnaires and included as a covariate in our linear regression models in order to account for potential differences in fat intake. It is plausible that diet contributes to circulating c9t11 CLA concentrations in a sex-specific manner; however, according to a recent study by Nikpartow et al., Canadian females have lower milk consumption than Canadian males [42]. And in an overview of Canadian eating habits, males were reported to consume more meat than females [43]. Thus, the significantly higher levels of CLA in females compared to males observed in this study has a low likelihood to be a result of differences in dietary intake of CLA.

In addition to diet, differential gene expression of *SCD1* can contribute to the differences observed in c9t11 CLA concentrations between males and females. Several hormones have been shown to affect *SCD1* expression [44]; thus, they could also contribute to sex differences. In that regard, further stratification of female population by HC use revealed that female users of HC had significantly higher circulating c9t11 CLA concentrations than females who were non-users in both Caucasian and Asian populations. Recently, an examination of the effect of HC use on proteomic biomarkers in plasma of TNH study participants revealed that while use of HC had a significant effect on alteration of proteomic biomarkers, the type of HC used and duration of use had no apparent effect. Thus, further stratification of our analysis according to type of HC used or duration of use was not performed in this study [45]. Plasma concentrations of c9t11CLA and 18:1 t11 were used to estimate D9D desaturation index. Although there was a trend towards greater [18:2c9t11/18:1 t11] desaturase indices in females using HC from both ethnicities, use of HC was not significantly associated with altered D9D desaturase index (Table 3). Several hormones have been shown to modulate *SCD1* expression. For instance, insulin has been shown to have a positive effect on *SCD1* transcription, while leptin and estrogen were shown to act as inhibitors [44]. Although estrogen is an inhibitor of *SCD1* expression, the effect of estrogen or hormonal contraceptives on circulating c9t11 CLA concentrations have not been previously reported. HC have been shown to increase levels of the polyunsaturated fatty acid docosahexaenoic acid in females using contraceptives compared to females that were not [30]. The positive association between HC and c9t11 CLA levels observed in this study warrants further research to elucidate the mechanism by which HC can increase circulating levels of c9t11 CLA. Future research by our laboratory will also investigate the effect of HC use on other D9D products such as 18:1n9 and 16:1n7.

To the best of our knowledge, association between *SCD1* SNPs and D9D activity, estimated by [18:2c9t11/18:1 t11] desaturase index, has not been previously reported. To determine whether *SCD1* SNPs contributed to the different levels of c9t11 CLA or to differences in D9D deasturase index between sexes, linear regression analyses were performed. Results revealed that rs10883463 was significantly (p = 0.0014) associated with altered D9D desaturation index in Caucasian males (Table 5). Of the Caucasian male participants in our study cohort, 17% had the genotype associated with decreased D9D desaturation index; however, linear regression models revealed no significant association between D9D indices and *SCD1* SNPs in the 3 additional populations tested (Caucasian females, Asian males and Asian females). The lack of additional associations between rs10883463 and other D9D indices (16:1/16:0 and 18:1/18:0) within the Caucasian male population led us to conclude that the association identified between this SNP and CLA may suggest a degree of specificity for 18:1 t11; however, this requires confirmation in other populations in order to substantiate this hypothesis. Furthermore, we acknowledge that this association was not significant following an adjustment for multiple testing, further reinforcing the need for independent verification. Nevertheless, in a recent study of variations of *SCD1* and associations with relative levels of palmitic and stearic acids in Caucasian and Asian subjects, Stryjecki et al. identified a significant association between *SCD1* SNP rs2060792 and lower palmitic acid but higher stearic acid levels in Caucasian women only; however, associations with [16:1/16:0] and [18:1/18:0] desaturase indices were not significant [28]. Our work in the present study, which has used a much larger cohort, supports this lack of association. The D9D activity is highest for 18:1/18:0, intermediate for c9t11/t11 and lowest for 16:1/16:0. While there is emerging evidence that *SCD1* polymorphisms are associated with either the precursor, product or desaturase indices of relevant fatty acids, there remains a need for direct evidence to establish these potential cause-effect relationships. Further validation is required to demonstrate the direct relationship between the SNP identified in this study (rs10883463) and SCD1 biochemistry.

In this study we acknowledge the limitation of using plasma as a source for fatty acid measurements, which typically reflects a combination of recent dietary intake and hepatic synthesis; however, since subjects fasted overnight prior to blood collection the contribution of dietary fat to the blood fatty acid profile is expected to be negligible. Although red blood cells or adipose tissue might be argued to better reflect long term status, Baylin et al. found that fasting plasma fatty acids correlated with fatty acids in adipose tissue [46]. However, there remains a need for more extensive studies to determine whether plasma, red blood cells, adipose tissue or other tissues are most suited for correlation analysis with *SCD1* polymorphisms. Another limitation to the present study was that food frequency questionnaire data for CLA did not correlate with plasma CLA values (data not shown), suggesting that the FFQ used in the present study may lack sensitivity with regards to estimating dietary CLA intake. Therefore we cannot fully negate the contribution of diet to plasma fatty acid levels. Future studies require careful measurement of CLA from multiple blood fractions in tandem with more extensive and detailed recording of dietary CLA intake. Nonetheless, results from this study provide fundamental knowledge regarding determinants of circulating CLA levels. Data demonstrate a minor role for genetic variation in *SCD1* in determining circulating CLA levels in different ethnicities or sexes and a significant role for hormonal contraceptive use in altering circulating CLA levels in females. These findings can be utilized in future clinical and nutritional studies when it is essential to understand factors that may have a significant influence on baseline levels of CLA (i.e. hormonal contraceptive use) versus factors that may have only a minor influence on CLA variation (i.e. genetic polymorphisms in *SCD1*).

## Conclusion

Overall, this study demonstrates that sex, HC use and *SCD1* polymorphisms can influence c9t11 CLA concentrations. The implications of CLA in chronic diseases such as obesity, inflammation, cardiovascular disease and cancer [47] necessitate better understanding of the diverse factors influencing circulating concentrations of this fatty acid as well as their sex- and ethnic-specific effects.

## Abbreviations

BMI: Body mass index; CLA: Conjugated linoleic acid; D9D: Delta-9 desaturase; HC: Hormonal contraceptives; HWE: Hardy-Weinberg equilibrium; MAF: Minor allele frequency; RA: Rumenic acid; SCD1: Stearoyl-CoA desaturase 1; SNP: Single nucleotide polymorphism; TFA: Trans fatty acid

## Competing interest

The authors declare that they have no competing interests.

## Authors’ contributions

SA performed data analysis, interpreted results, assisted with gas chromatography and drafted the manuscript. SC carried out gas chromatography analyses and critically revised manuscript. JW assisted in data analysis. KR carried out genotyping studies. DN collected DNA samples from all participants. AB, AE and DMM assisted in interpretation of results and critically revised manuscript. DWLM conceived and designed study, assisted in interpretation of data and critically revised manuscript. All authors read and approved the final manuscript.
